# The Efficacy of Hypnotherapy in Reducing Burnout and Related Psychopathologies: Protocol for a Systematic Review and Meta-Analysis

**DOI:** 10.2196/69733

**Published:** 2026-07-17

**Authors:** Santosha Veeramachaneni, Elizabeth Park, Jeffrey Martin, Marc Ringor, Gregory Brown, Anne Weisman, Kavita Batra

**Affiliations:** 1 Department of Medical Education, Kirk Kerkorian School of Medicine at UNLV, Las Vegas, NV, United States; 2 Department of Behavioral Health and Psychiatry, Kirk Kerkorian School of Medicine at UNLV, Las Vegas, NV, United States; 3 Department of Internal Medicine, Kirk Kerkorian School of Medicine at UNLV, Las Vegas, NV, United States; 4 Office of Well Being and Integrative Medicine, Kirk Kerkorian School of Medicine at UNLV, Las Vegas, NV, United States; 5 Office of Research and Department of Medical Education, Kirk Kerkorian School of Medicine at UNLV, 625 Shadow Ln, Las Vegas, NV, 89106, United States, 1 (702) 895-3011

**Keywords:** hypnosis, hypnotherapy, burnout, depression, anxiety

## Abstract

**Background:**

Hypnosis is a focused state of consciousness that enhances concentration, attention, and responsiveness to suggestion. It has shown efficacy in treating psychiatric disorders such as depression, anxiety, and posttraumatic stress disorder (PTSD). However, its potential for addressing burnout and related symptoms remains underexplored. This systematic review and meta-analysis aims to assess the effectiveness of hypnotherapy in alleviating symptoms of burnout and associated conditions, including depression, anxiety, and PTSD.

**Objective:**

This study aims to evaluate the efficacy of hypnotherapy in treating burnout and its related psychopathologies, including depression, anxiety, and PTSD.

**Methods:**

A comprehensive search was conducted across PubMed, Scopus, and PsycINFO for peer-reviewed, English-language observational and experimental studies published up to February 2024. Only studies with adults (>18 y) who received hypnotherapy for psychiatric symptoms will be included. Data extraction will focus on treatment effects related to burnout and associated psychiatric conditions. Statistical analysis will be performed using Comprehensive Meta-Analysis (version 4.0). A random-effects model will be used to account for heterogeneity, with Cochran *Q* and *I*² statistics used to assess variability. Subgroup analyses will explore moderators such as sociodemographic factors, country, and study quality. Sensitivity analyses will identify influential studies, and publication bias will be assessed using funnel plots and the Egger test. All analyses will be 2-sided (*P*<.05), and the results will be presented in forest plots.

**Results:**

The search strategy underwent a PRESS (Peer Review of Electronic Search Strategies) review in December 2023 and was registered in PROSPERO (International Prospective Register of Systematic Reviews) in January 2024. Database searches were completed between February and April 2024, followed by title and abstract screening from May to November 2024. Full-text screening began in December 2024, with data extraction conducted from January through April 2025. A preliminary narrative synthesis was completed between May and August 2025. Quantitative analysis began in September 2025 and is ongoing, with completion anticipated in early summer 2026. The final findings are expected to be submitted for publication by fall 2026.

**Conclusions:**

This review aims to synthesize existing evidence on the potential role of hypnotherapy for burnout and related psychiatric symptoms. This systematic review will provide an updated synthesis of evidence on hypnotherapy for burnout and associated psychological outcomes. Limitations include variability in study designs and measurement tools. The results will be disseminated through peer-reviewed journals and scientific meetings to guide future clinical and research applications

## Introduction

Burnout, characterized by exhaustion, cynicism, and inefficacy, is a pervasive form of job-related stress that significantly impacts work efficiency and overall well-being [1-4]. Particularly critical in the health care sector, burnout among nurses and physicians not only diminishes their productivity and job satisfaction but also adversely affects patient care, leading to increased medical errors and reduced patient satisfaction [5,6]. This issue extends beyond health care professionals, as burnout is prevalent among medical students, with nearly half reporting symptoms, highlighting the need for early intervention [7]. Burnout is not classified as a mental disorder but rather as an occupational syndrome characterized by emotional exhaustion, depersonalization, and a diminished sense of personal accomplishment. It develops from chronic workplace stress that has not been successfully managed and is influenced by both individual and organizational factors, including excessive workload, insufficient resources, role ambiguity, and limited managerial or peer support. Although burnout frequently coexists with psychological conditions such as depression, anxiety, and posttraumatic stress disorder (PTSD), it represents a distinct construct with unique etiological and intervention pathways. Research has demonstrated a positive correlation between burnout and depression, indicating that the emotional exhaustion characteristic of burnout aligns closely with symptoms of major depressive disorder [8-11]. Similarly, the cynicism dimension of burnout can manifest as irritability and a negative attitude, which are commonly associated with depressive moods. Anxiety also plays a significant role in the burnout process, influencing feelings of inefficacy and exhaustion [3]. Studies suggest that anxiety not only exacerbates burnout but may also serve as a stable trait that influences its progression over time. Furthermore, PTSD shares characteristics with burnout, particularly in the realms of emotional exhaustion and depersonalization. Evidence shows a significant relationship between burnout symptoms and PTSD, particularly in high-stress professions such as firefighting and nursing [12,13].

Given these complex interconnections, it is clear that burnout does not exist in isolation. Its symptoms often overlap with those of depression, anxiety, and PTSD, suggesting a need for comprehensive approaches to treatment [14-17]. Hypnosis is defined as a focused state of consciousness characterized by absorbed attention, reduced peripheral awareness, and an increased capacity to respond to suggestions. This state may be induced through verbal guidance, relaxation techniques, or imagery-based instructions [14]. Hypnotherapy, in contrast, refers to the structured clinical application of hypnosis by a trained practitioner for therapeutic purposes. Hypnotherapy incorporates hypnotic induction followed by targeted therapeutic suggestions or interventions aimed at modifying thoughts, emotions, or behaviors relevant to the presenting condition. Therapeutically, hypnotherapy is thought to exert its effects through several mechanisms, including enhanced attentional control, increased receptivity to adaptive suggestions, modulation of cognitive-emotional processing, and activation of neural pathways involved in self-regulation and stress response. These mechanisms may be particularly relevant for addressing burnout and related psychological symptoms, which involve disruptions in emotional regulation, cognitive load, and stress reactivity [14].

Hypnosis has also been shown to be effective in reducing anxiety, particularly in specific populations such as patients with cancer and individuals preparing for surgery [18]. Its ability to create a relaxed state of mind helps patients redirect their focus away from anxiety-inducing stimuli, which may be particularly beneficial for health care professionals and students experiencing burnout. Additionally, hypnosis has demonstrated promise in addressing PTSD symptoms by allowing individuals to access traumatic memories in a controlled manner, thereby facilitating emotional processing and improving sleep quality [19]. Notably, both hypnosis and self-hypnosis have garnered attention as potential interventions for burnout. Studies suggest that self-hypnosis may alleviate burnout symptoms by promoting compassion satisfaction and enhancing job engagement [20]. A study involving anesthesiologists revealed that participation in a medical hypnosis training program resulted in significant improvements across all 3 dimensions of burnout [21]. Furthermore, self-hypnosis has shown effectiveness in reducing stress levels among medical students preparing for examinations, indicating its potential as a preventative measure against burnout [22].

Despite our updated literature search extending through 2025, no recent randomized controlled trials or systematic reviews were identified that specifically evaluate hypnotherapy for burnout. While contemporary studies have investigated hypnosis for related psychological outcomes such as stress, anxiety, and depression, controlled evidence targeting occupational burnout remains limited. This absence of recent randomized controlled trials (RCTs) itself underscores a critical gap in the literature and highlights the timeliness and necessity of this review. The limited empirical evidence such as the quasi-experimental study by Boselli et al [21] demonstrating reduced burnout symptoms following hypnosis training further reinforces the need for a systematic synthesis that maps available studies, appraises methodological rigor, and identifies directions for future clinical trials.

To clarify the need for this review, we conducted a focused appraisal of prior systematic reviews and meta-analyses examining hypnosis and related interventions for psychological outcomes. Table 1 summarizes existing evidence syntheses, their key findings, and the gaps our review aims to address.

As summarized in Table 1, prior reviews primarily addressed stress, depression, anxiety, or PTSD separately, often excluding burnout as a primary construct. No existing review has comprehensively evaluated hypnotherapy across these interrelated psychopathologies within occupational contexts. This gap underscores the need for a systematic synthesis focused on burnout and its comorbid psychological outcomes. Given the intricate relationships between burnout and other psychopathologies, this review aims to systematically examine the literature on the effects of hypnosis on burnout as the primary outcome, with depression, anxiety, and PTSD evaluated as secondary outcomes. Because these psychological conditions frequently co-occur with burnout and may influence its severity or responsiveness to intervention, they are examined as secondary outcomes that may moderate or contextualize the effects of hypnotherapy on burnout. To the authors’ best knowledge, this will be the first systematic review specifically focusing on how hypnosis can be used to alleviate symptoms across these interconnected mental health challenges. By addressing this gap, the review aims to provide insights that may be particularly relevant for populations experiencing high levels of occupational stress, such as health care workers and students, while recognizing that burnout and related psychological symptoms also affect the broader adult population.

**Table 1.** Summary of existing reviews and identified gaps.

Author, year	Focus or population	Design or scope	Main findings	Key limitations or gaps
Fisch et al [23], 2017	Hypnosis for stress management (general population)	Systematic review	Hypnosis effective in reducing perceived stress	Did not assess burnout or compare psychiatric outcomes
Milling et al [15], 2019	Hypnotic interventions for depression	Meta-analysis	Hypnosis significantly improves depressive symptoms	Excluded burnout, no occupational stress analysis
Valentine et al [16], 2019	Hypnosis for anxiety	Meta-analysis	Hypnosis effective for anxiety management	No subgroup analysis for burnout-related stressors
Rotaru and Rusu [17], 2016	Hypnotherapy for PTSD^a^	Meta-analysis	Hypnosis beneficial in PTSD symptom reduction	Focused solely on trauma; excluded burnout
Pang et al [22], 2024	Hypnotherapy as treatment for depression	Scoping review	Emerging evidence for depression	No integration with occupational burnout or co-occurring disorders
Kern et al [24], 2024	Psychotherapeutic interventions for burnout	Umbrella review	Synthesized CBT^b^, mindfulness, and relaxation therapies	Hypnosis not included; limited evidence for health care professionals.
Boselli et al [21], 2021	Hypnosis training for health care providers	Survey study	Reduced burnout in anesthesiologists after hypnosis training	Observational design, small sample size
Ruysschaert [20], 2009	Self-hypnosis for burnout prevention	Conceptual paper	Theoretical mechanisms for compassion fatigue prevention	Narrative only, no quantitative synthesis
Wolf et al [25], 2022	Neurobiological changes in hypnosis	Systematic review	Identified brain activity modulation via hypnosis	Not condition-specific; no occupational application

a PTSD: posttraumatic stress disorder.

b CBT: cognitive behavioral therapy.

## Methods

### Ethical Considerations

As this study does not involve the direct involvement of human participants, an institutional ethical review will not be warranted. However, to adhere to a strict methodology, the protocol of this systematic review and meta-analysis has been registered on PROSPERO (International Prospective Register of Systematic Reviews; CRD42024497817, registered on January 23, 2024). The record details can be seen at [26]. PROSPERO provides a unique permanent registration number to the protocol that prevents duplication, thereby reducing reporting bias.

### Review Question

This review aims to address the following questions. The primary question is what the effect of hypnosis or hypnotherapy is on burnout symptoms among adults. The secondary questions are as follows: (2) What is the effect of hypnosis or hypnotherapy on associated psychological outcomes, specifically depression, anxiety, and PTSD? (3) How do study characteristics (eg, population, intervention format, session frequency and duration, delivery modality) influence the effectiveness of hypnotherapy for burnout and related psychological outcomes? (4) What methodological factors contribute to variations in findings across the included studies?

### Inclusion and Exclusion Criteria

The PECOS (Population, Exposure, Comparison, Outcome, Study Design) framework (Textbox 1) was used to develop the eligibility criteria for this systematic review. Eligible studies included adult participants (18 years or older), regardless of occupation or geographic location. Although health care workers and students represent key populations of interest due to the high prevalence of burnout, the review is not restricted to these groups. Only peer-reviewed studies published in English were included due to resource constraints, and no geographical restrictions were applied.

Textbox 1. **PECOS (Population, Exposure, Comparison, Outcome, Study design) framework for the eligibility criteria of the studies used in this review (Comparators are not listed due to the heterogeneity in study designs; many eligible studies do not include a defined control group).**

P: Adult participants aged 18 years and older, who have received hypnosis for psychological morbidities

E or I: Hypnosis or hypnotherapy

C: None

O: Changes in severity or presentation of psychological symptoms, such as anxiety, depression, stress, burnout, and sleep problems.

S: Case series, case control, prospective cohort, randomized controlled trials, and other observational designs

Study designs included in this systematic review and meta-analysis are as follows: RCTs, case-control studies, cohort studies, case series, and other observational and experimental studies. Observational designs were included because the evidence base for hypnotherapy in the context of burnout is still limited, and many available studies use pre-post or cohort designs. Including these studies enables a more complete synthesis of the existing literature, while the application of design-specific National Heart, Lung, and Blood Institute (NHLBI) quality thresholds ensures that only methodologically sound observational studies contribute to the quantitative analysis. Studies that were excluded are as follows: reviews, duplicates, commentaries, opinions, letters to the editor, position papers, and gray literature. We have not specified a time frame criterion, as hypnosis in psychiatry has been documented as a treatment modality for hundreds of years, and it was important to include all eligible historical studies in our analysis. Additionally, comparators were not prespecified because eligible studies include a range of designs, including pre-post single-group studies, waitlist controls, and treatment-as-usual comparators. Due to this heterogeneity, specifying a single comparator category in the PECOS framework would not accurately represent the available evidence. While a range of study designs was included to comprehensively map the evidence, only those meeting predefined criteria for methodological rigor and suitability for evaluating intervention effects were included in the quantitative synthesis, as detailed in the “Statistical Plan” and “Quality or Risk of Bias Assessment” sections.

### Informational Sources and Search Strategy

A systematic search of bibliographical electronic databases was conducted on all English-language human studies. Articles were not excluded based on the date of publication. A comprehensive search and article retrieval strategy was planned by the study’s investigators, guided by a library subject matter expert, to find potentially relevant articles in the following databases: PubMed, Scopus, and PsycINFO. In accordance with the search development and optimization method proposed by Bramer et al [27], the search strategy was developed in PubMed and then translated into the syntax of Scopus and PsycINFO [1]. The search strategy was optimized for each database using a thesaurus, free-text search terms, as well as MeSH terms. Informed by the PECOS or PICOS (Population, Intervention, Comparison, Outcome, Study Design) framework, search strings for each database were developed iteratively and revised as new search terms were discovered. In consultation with a subject matter expert, the search strategy was finalized based on the PRESS (Peer Review of Electronic Search Strategies) 2015 Evidence-Based Checklist [2]. The following search terms were used: “‘stress,” “depression,” “anxiety,” “Hamilton Anxiety Scale,” “HAM-A”, “Hamilton Depression Rating Scale,” “HAM-D,” “Beck Depression Inventory,” “Patient Health Questionnaire,” “PHQ-9,” “self-hypnosis,” “hypnosis,” “hypnotherapy,” “anxiety disorders,” “anxiety management,” “anxiety screening,” “anxiety sensitivity,” “Depression Screening,” “Major Depression,” “stress,” “stress and coping measures,” and “stress management.” A combination of appropriate Boolean operators (AND, OR), truncation, and the MeSH terms was used. Table 2 includes the full search strategy in detail.

**Table 2.** Search strategy used in this review.

Databases	Search strings	Inclusion or exclusion	Number of articles	Total number of articles in search
PubMed				
1	(self-hypnosis OR hypnosis [MH] OR hypnotherapy)	Inclusion: English	16,366	—^a^
2	(stress OR depression OR anxiety OR Hamilton Anxiety Scale OR HAM-A OR Hamilton Depression Rating Scale OR HAM-D OR Beck Depression Inventory OR Patient Health Questionnaire OR PHQ-9 OR psychological symptoms OR psychiatric symptoms OR mental health OR burnout OR sleep OR insomnia OR dysphoria OR mood OR post-traumatic stress disorder OR bipolar)	Inclusion: English	3,747,069	—
3	1 AND 2	Exclusion: books and documents	—	4976
Scopus				
1	(self-hypnosis OR hypnosis OR hypnotherapy)	Inclusion: English	55,176	—
2	(stress OR depression OR anxiety OR Hamilton Anxiety Scale OR HAM-A OR Hamilton Depression Rating Scale OR HAM-D OR Beck Depression Inventory OR Patient Health Questionnaire OR PHQ-9 OR psychological symptoms OR psychiatric symptoms OR mental health OR burnout OR sleep OR insomnia OR dysphoria OR mood OR post-traumatic stress disorder OR bipolar)	Inclusion: English	653,218	—
3	1 AND 2	Inclusion: article, review	—	7539
PsychINFO				
1	(DE “Hypnosis” OR DE “Hypnotherapy”)	Inclusion: English	12,538	—
2	(DE “Major Depression” OR DE “Depression (Emotion)” OR DE “Depression Screening” OR DE “Affective Disorders” OR DE “Anxiety” OR DE “Anxiety Disorders” OR DE “Anxiety Screening” OR DE “Stress” OR DE “Stress Management” OR DE “Stress and Coping Measures” OR DE “Burnout” OR DE “Sleep Wake Disorders” OR DE “Insomnia” OR DE “Bipolar Disorder” OR DE “Bipolar I Disorder” OR DE “Bipolar II Disorder” OR DE “Posttraumatic Stress Disorder” OR DE “Psychiatric Symptoms” OR DE “Mental Health”)	Inclusion: English	526,748	—
3	1 AND 2	Inclusion: academic journal	—	983

a Not applicable.

### Screening

All references were imported into the systematic review tool Rayyan for screening [28]. After deduplication, all references were assessed by 2 independent reviewers to check for inclusion and exclusion criteria. Titles, abstracts, and full texts of articles were screened in a systematic and sequential manner.

### Data Extraction and Main Data Elements

Two reviewers independently extracted the relevant data elements from the eligible full texts of the articles and recorded these variables in a standardized code book. A double extraction method was used to ensure accuracy and completeness. After data extraction, disparities were resolved by consensus and discussion with a third reviewer or a “tie-breaker.” Attempts to contact the corresponding authors of the included articles will be made if more information about the individual study data is needed. However, since this is a time-bound project, such an attempt will only be made within a window of 4 weeks. If author contact attempts are unsuccessful within this timeframe, analyses will proceed using available data, and sensitivity analyses will be conducted to assess potential bias. Because the review team does not have resources for professional translation, the selection of studies is limited to English-language publications.

The following data elements were extracted: author name, publication year, study country, study design, time point of data collection, recruitment method, type of survey tool used and scoring criteria, sample size, participants’ characteristics (eg, age, gender, sexual orientation, gender identity), intervention type, and effect on psychological morbidities. For studies reporting multiple measures for the same psychological construct, a predefined hierarchy of instruments was applied to maintain consistency in the meta-analysis. For burnout, the Maslach Burnout Inventory (MBI) was prioritized; for depression, the Hamilton Depression Rating Scale (HAM-D); for anxiety, the Hamilton Anxiety Rating Scale (HAM-A); and for PTSD, the Clinician-Administered PTSD Scale (CAPS), where available. When several validated tools are reported within a study, the primary outcome specified by the authors or the measure with the most complete data and largest sample size was selected for quantitative synthesis.

Additional data will be extracted to characterize how the hypnotherapy intervention was delivered, including the number and length of sessions, total treatment duration, provider type (eg, licensed clinician, certified hypnotherapist, or psychologist), delivery modality (in-person, virtual, individual, or group format), and intervention setting (eg, clinical, occupational, or academic). These elements will allow for comparison across differing implementation approaches. Primary and secondary outcomes will be extracted using standardized tools, with burnout prioritized using the MBI, and depression, anxiety, and PTSD assessed using validated measures such as the HAM-D, HAM-A, and CAPS, respectively.

### Quality or Risk of Bias Assessment

**Figure 1.**
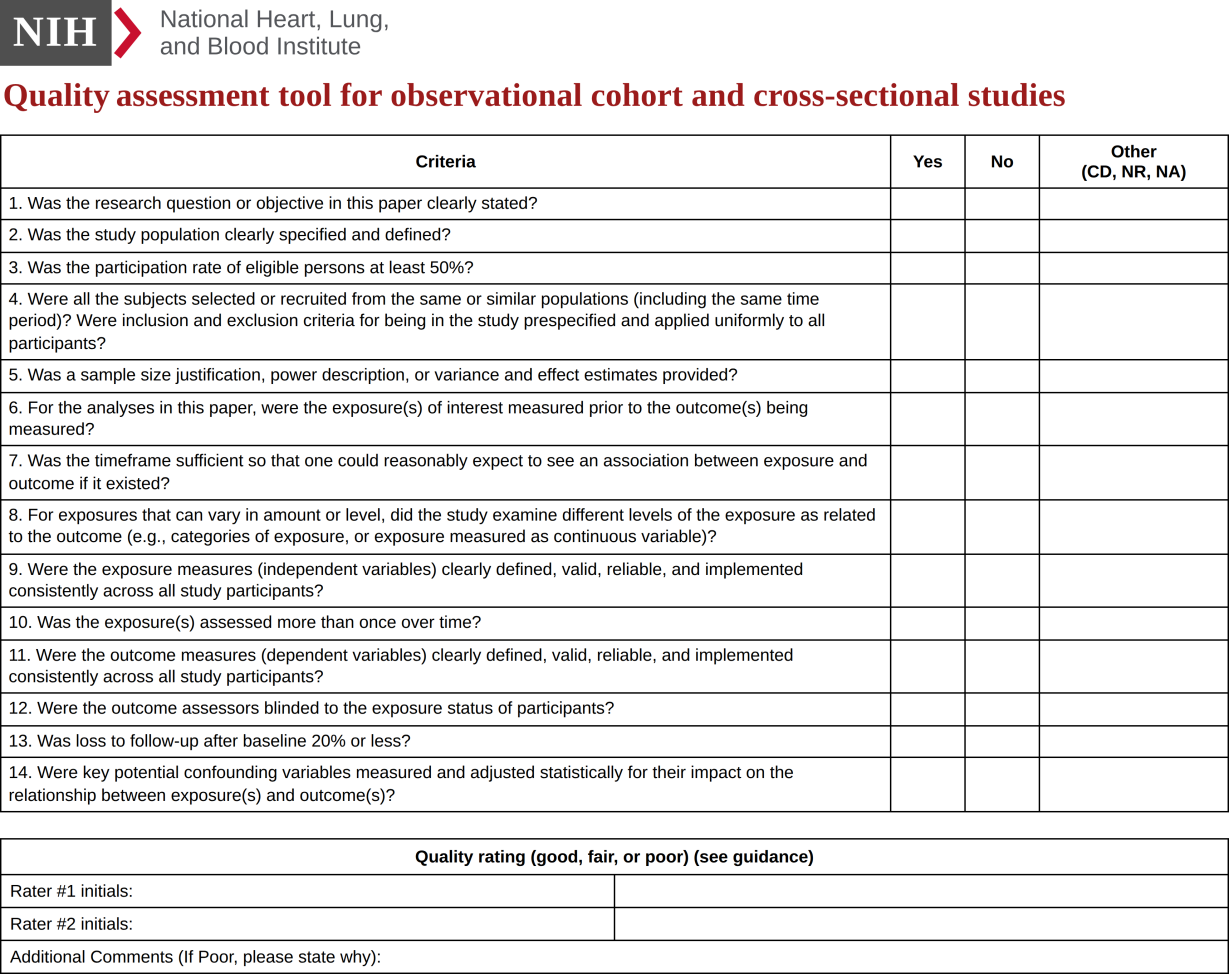
National Heart, Lung, and Blood Institute’s study quality assessment tool. CD: cannot determine; NA: not applicable; NR: not reported.

For the quality assessments of the included studies, the NHLBI quality assessment tool will be used [29]. Two reviewers will assess the quality of the full texts and will perform scoring independently. The Kappa statistics will be calculated to measure the interrater agreement. The NHLBI tool has 14 items in the checklist to evaluate all essential components of original research studies. Quality will be rated as poor (0‐4 out of 14 questions), fair (5‐10 out of 14 questions), or good (11‐14 out of 14 questions) as guided by the tool (Figure 1). The NHLBI quality assessment tool will be applied using the design-specific checklists corresponding to each study type (eg, separate tools for RCTs, cohort, case-control, and cross-sectional studies). Each study will be evaluated within its methodological context, and bias domains including selection, performance, detection, and reporting biases will be assessed accordingly. Interrater agreement will be calculated using Cohen κ to ensure scoring consistency. Scores will not be combined across study types; instead, quality will be interpreted qualitatively to guide inclusion in the meta-analysis or narrative synthesis. Because cross-sectional studies cannot establish treatment effectiveness, studies using this design will be included only for descriptive or contextual purposes and will not contribute to the quantitative meta-analysis. Quality assessments will guide analytic decisions: only studies rated as “fair” or “good” and using designs appropriate for evaluating intervention effects (eg, RCTs, quasi-experiments, longitudinal pre-post studies) will be included in the pooled analysis. Studies rated as “poor” quality or those whose design precludes causal inference will be synthesized narratively. Studies will be eligible for inclusion in the quantitative meta-analysis only if they use designs capable of evaluating intervention effects, including RCTs, quasi-experimental studies, and longitudinal pre-post studies, and are rated as fair or good quality based on the NHLBI assessment tools. Studies with designs that do not support causal inference, such as cross-sectional studies, case series, or studies rated as poor quality, will not be included in the meta-analysis and will instead be synthesized narratively. In addition, meta-analysis will be conducted only when at least 3 studies are sufficiently comparable in terms of population, intervention, and outcome measures.

### Statistical Plan

First, the results of all finally included studies will be described succinctly in the form of a summary table. The pooled estimates with a 95% CI will be generated using the Comprehensive Meta-Analysis Package (version 4.0). Effect sizes will be calculated based on study design and outcome type. For continuous outcomes (eg, burnout, depression, anxiety, and PTSD scores), standardized mean differences (Hedges *g*) with 95% CIs will be computed. For studies with pre-post designs without a control group, within-group effect sizes will be calculated using standardized mean change. For randomized and controlled studies, between-group effect sizes will be derived by comparing intervention and comparator groups at postintervention. If dichotomous outcomes are reported, pooled estimates will be calculated using odds ratios. When multiple outcome measures are reported for the same construct, a predefined hierarchy of validated instruments will be applied, and the primary outcome identified by the study authors or the measure with the most complete data will be selected. If multiple time points are available, postintervention outcomes will be prioritized for the primary analysis. Statistical heterogeneity will be assessed using the Cochran Q test and quantified using the *I*² statistic, with thresholds of low (25%), moderate (50%), and high (75%) heterogeneity. In the presence of substantial heterogeneity, subgroup and sensitivity analyses will be conducted to explore potential sources, including study design, population characteristics, intervention format, and study quality. A random-effects model will be used to calculate pooled estimates, as this is a more robust estimate regardless of heterogeneity. Cochran Q and *I*² statistics will be used as indicators of heterogeneity. If enough studies are available in subgroups, further analyses based on moderator variables, such as sociodemographic variables, country, and study quality, will also be conducted. Sensitivity analysis will be conducted to identify studies that may severely affect the pooled prevalence. A funnel plot and Egger linear regression test will be used to assess publication bias. The significance level will be set as 2-sided, with *P*<.05. Forest plots will be used to present the data. In the meta-analysis of factors, variables common to all participants will be pooled quantitatively to examine the effect of the intervention on various psychopathologies. If the data do not allow for quantitative pooling, for example, due to substantial clinical or methodological heterogeneity, incompatible outcome measures, or an insufficient number of comparable studies, a narrative synthesis will be conducted. This synthesis will summarize the direction and magnitude of effects, study-level characteristics, intervention parameters, and quality ratings. Patterns across studies will be explored using structured comparisons by population, intervention format, and outcome type. This approach ensures that meaningful conclusions can still be drawn even when a meta-analysis is not possible.

### Interim Checkpoints and Protocol Amendments

Progress checkpoints were conducted at key stages following title and abstract screening, after the completion of full-text screening, and prior to data analysis to ensure adherence to the planned protocol and timely progress. Any significant methodological changes or deviations from the initial protocol were documented and submitted as amendments to the PROSPERO registration, and these updates will be transparently reported in the final study.

## Results

**Figure 2.**
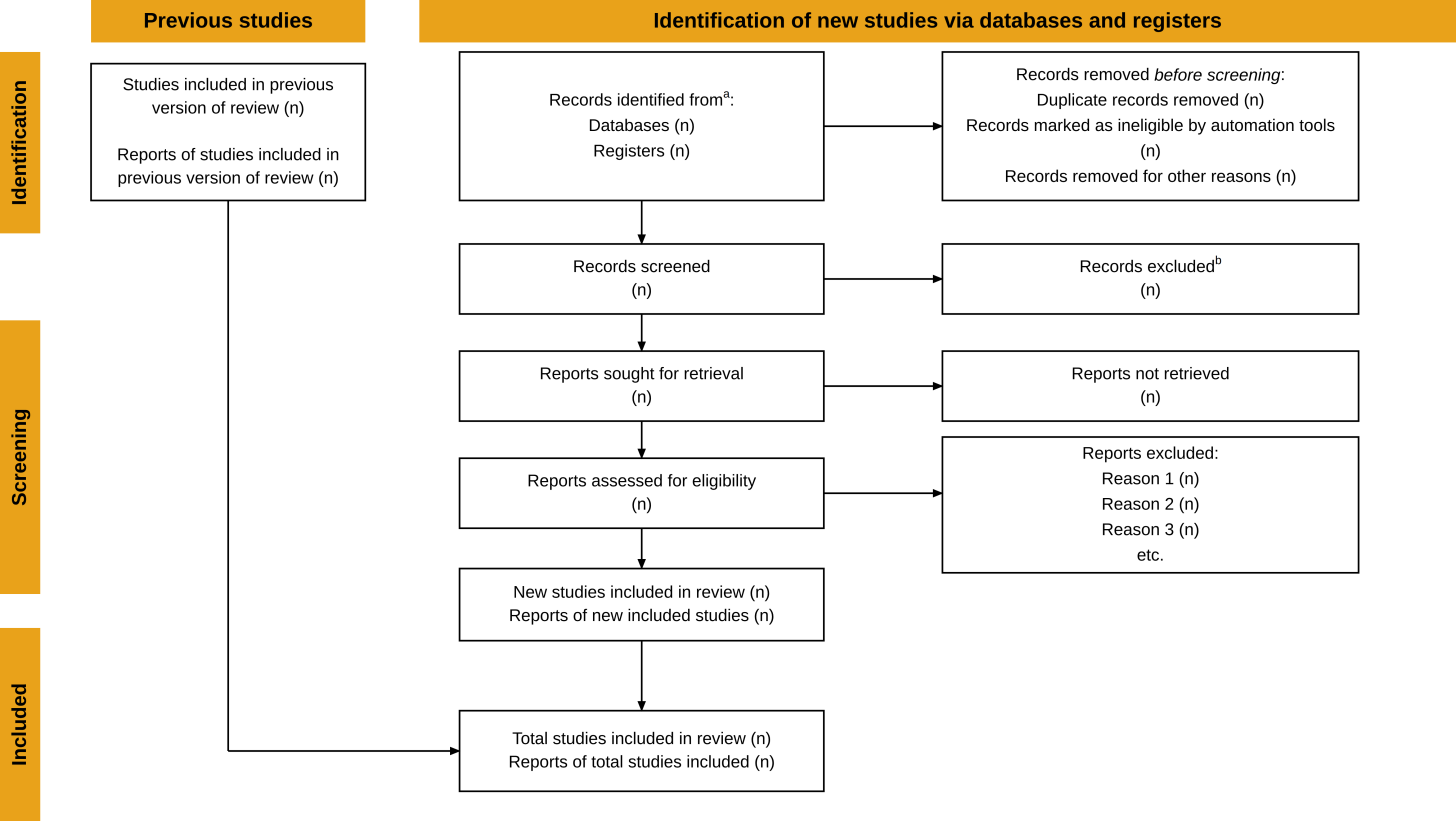
PRISMA (Preferred Reporting Items for Systematic Reviews and Meta-Analyses) diagram. ^a^Consider, if feasible to do so, reporting the number of records identified from each database or register searched (rather than the total number across all databases/registers). ^b^If automation tools were used, indicate how many records were excluded by a human and how many were excluded by automation tools.

During the screening process, all reasons for exclusion were documented at each step of the screening. A PRISMA (Preferred Reporting Items for Systematic Reviews and Meta-Analyses) flow diagram will be used for describing the study selection process (Figure 2).

**Figure 3.**
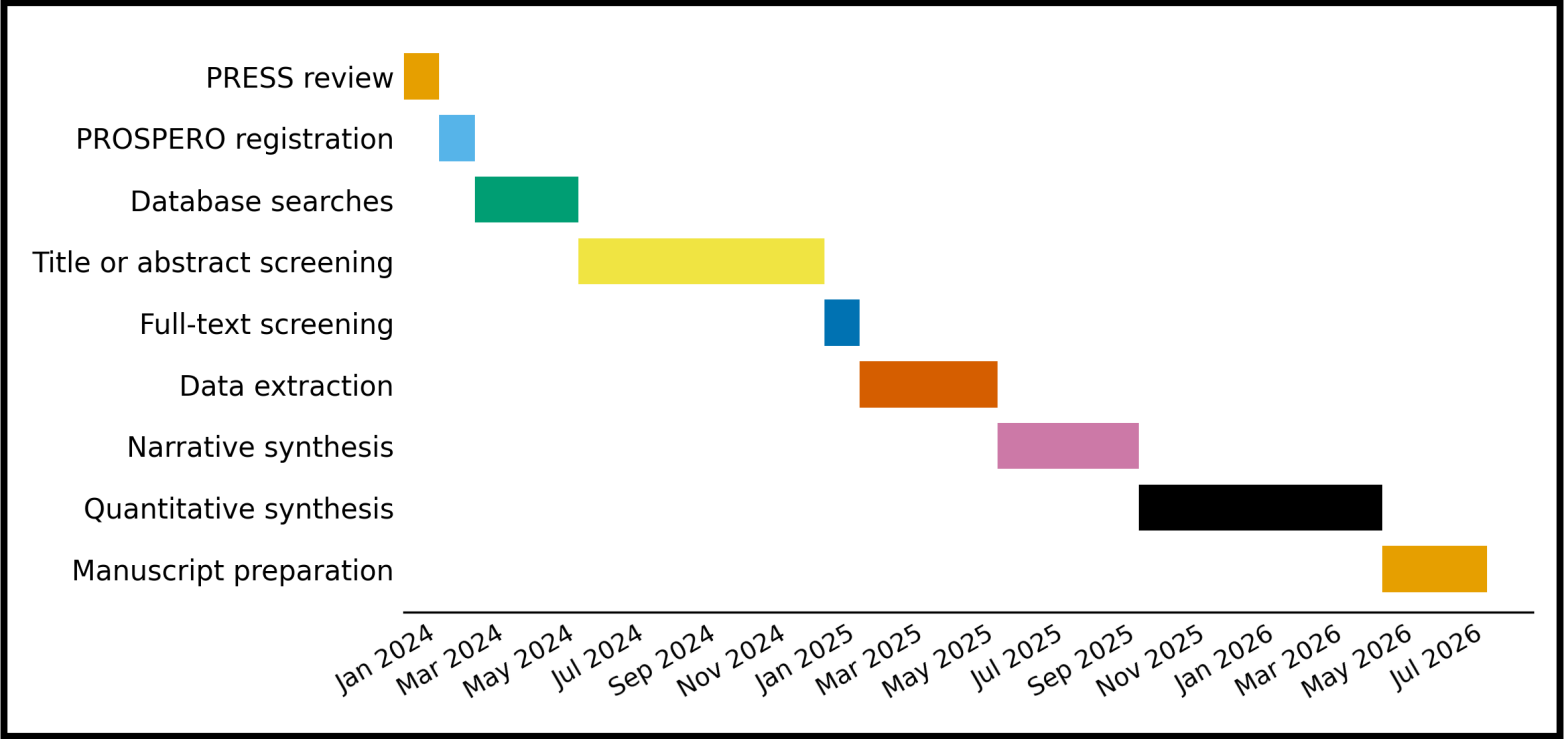
Gantt chart of systematic review timeline. PRESS: Peer Review of Electronic Search Strategies; PROSPERO: International Prospective Register of Systematic Reviews.

The search strategy underwent a PRESS review with academic librarians in December 2023, followed by PROSPERO registration in January 2024. Database searches and article retrieval were completed between February and April 2024. Title and abstract screening began in May 2024 and continued through November 2024. Full-text screening commenced in December 2024, with data extraction conducted from January to April 2025. A preliminary narrative synthesis was completed between May and August 2025. Quantitative synthesis began in September 2025 and is ongoing, requiring extensive data harmonization; it is expected to be completed in early summer 2026. The findings will be organized, synthesized, and submitted for publication in a peer-reviewed journal by early fall 2026. The purpose of this project is to provide a comprehensive analysis and enhance the understanding of the effectiveness of hypnosis in treating psychological morbidities. This section reflects the procedural status of the project only; no analytical results or findings are presented. A timeline is provided in Figure 3.

## Discussion

### Principal Findings

This systematic review and meta-analysis is anticipated to demonstrate that hypnotherapy may have beneficial effects in reducing symptoms of burnout and related psychopathologies, including depression, anxiety, and PTSD. While the strength and consistency of the evidence will depend on study quality and design, we expect to observe trends suggesting positive therapeutic outcomes, particularly in health care populations and students experiencing occupational stress. Building upon prior work that has primarily focused on stress, depression, or anxiety individually, this review aims to provide an integrated synthesis addressing the intersection between burnout and these related conditions. The study is designed to fill the evidence gap regarding the role of hypnosis as a distinct therapeutic modality for burnout, distinguishing it from mindfulness and other relaxation-based interventions.

Although hypnosis has been a therapeutic tool for centuries, to date, there have been very few systematic reviews regarding its use in the treatment of psychiatric pathologies. While the use of hypnotherapy in clinical settings has recently gained resurgence, the practice has been subject to controversy surrounding its efficacy and impacts on patients with psychiatric conditions [30]. Hypnosis differs from interventions such as cognitive behavioral therapy and mindfulness due to its focus on inducing a state of increased suggestibility, which allows the patient to experience an altered state of agency. As a result, subsequent changes to thoughts, actions, and symptoms feel involuntary and outside the patient’s control [31]. Our study aims to conduct a comprehensive systematic review of the currently available literature investigating the efficacy of hypnotherapy for burnout, stress, depression, anxiety, and other psychiatric symptoms. Although there have been studies and systematic reviews regarding hypnosis as a treatment, their focus is on its impacts on pain, physical health, and mood disturbances secondary to illness or hospitalization [32]. Limited reviews have been conducted regarding the use of hypnosis to treat psychopathology symptoms, and none have focused on the treatment of burnout [23,33]. Our study focuses on the efficacy of hypnosis in treating primary psychiatric illness and symptoms in relation to burnout, an area of research that still requires further investigation.

Burnout in physicians, including trainees, is a prevalent issue, with studies finding 45% to 55% of physicians and medical students reporting at least one symptom of burnout [7,34]. Furthermore, burnout can be considered an occupational hazard and is shown to diminish health outcomes for both health care providers and patients [35-38]. Burnout’s comorbidity with psychopathologies such as depression, anxiety, and PTSD increases strain on the health care system, as the experience of burnout can perpetuate symptoms of other disorders [39,40]. Thus, addressing this growing crisis is imperative to maintaining the integrity of the health care workforce and improving care for patients. Current research in burnout prevention and treatment primarily focuses on interventions such as cognitive behavioral therapy and mindfulness [24,41]. Although prior systematic reviews have explored the effects of hypnosis on stress, depression, and anxiety, none have systematically synthesized evidence on burnout as a primary construct. As outlined in our gap analysis (Table 1), previous reviews largely excluded burnout or its occupational correlates. Therefore, this review represents the first comprehensive effort to evaluate hypnotherapy’s impact on burnout and its associated psychopathologies, integrating these interconnected outcomes to inform future clinical and research directions. Examining the impact of hypnosis and hypnotherapy on burnout symptoms can provide a valuable avenue for the identification, prevention, and treatment of burnout in health care professionals.

### Strengths and Limitations

One strength of our review is the breadth of psychiatric pathology included in our investigation. While current studies primarily focus on the efficacy of hypnotherapy on specific psychiatric illnesses, our comprehensive study will allow us to aggregate the data and directly compare the effects of hypnotherapy on different symptoms and pathologies. This will better allow clinicians to understand which conditions may be more responsive to hypnotherapy. Additionally, many of the current studies regarding the use of hypnosis and hypnotherapy consolidate it with similar techniques, such as mindfulness meditation or guided imagery. We created an operational definition of hypnosis and hypnotherapy, and thus our review will be able to better distinguish and isolate the use and impacts of hypnosis as a treatment. Furthermore, we did not impose a time limit on the studies we included, thereby ensuring that historical data on the use of hypnosis were also considered. This approach allowed for a robust and thorough understanding of hypnosis as a stand-alone therapeutic intervention across a wide range of psychiatric conditions.

Our review’s focus on the larger-scale impacts of hypnosis on psychiatric pathology presents certain limitations. Primarily, the lack of a comparison treatment or placebo group makes it difficult to quantify the effects of hypnotherapy, especially in contrast to current gold-standard treatments. Additionally, due to the breadth of psychiatric illnesses we plan to include in our review, our study will not investigate the specific effects of hypnosis on individual pathologies, such as its impact on mania versus depression in bipolar disorder or its efficacy in different types of depressive disorders. Excluding related therapeutic modalities like guided meditation, visualization, and autogenic training based on our operational definition of hypnosis, may exclude beneficial techniques that share similarities with hypnosis [42]. Furthermore, our focus on individuals with primary psychiatric disorders, while excluding those with significant comorbidities or psychiatric symptoms secondary to other conditions (eg, surgical anxiety or depressed mood due to chronic pain or illness), limits the generalizability of our findings. This exclusion may prevent us from understanding the full potential of hypnosis across a broader range of psychological and psychosomatic conditions, indicating areas where further research is necessary. Additionally, this review is limited to English-language publications due to resource constraints, which may exclude relevant evidence published in other languages. These weaknesses present opportunities for further research. The inclusion of multiple psychological outcomes and diverse study designs may introduce statistical heterogeneity, which could limit the comparability of the findings across studies. To address this, subgroup and sensitivity analyses will be conducted to identify and explore sources of heterogeneity. Nevertheless, variations in study populations, intervention formats, and measurement tools may complicate the interpretation of pooled results and should be considered when evaluating the overall conclusions.

Our systematic review will provide a comprehensive understanding of the efficacy of hypnotherapy techniques in treating psychiatric illnesses. As a nonpharmaceutical treatment approach, hypnotherapy may be a cost-effective tool with a low physical side effect profile, which can easily be performed as an adjunctive treatment without the risk of negative pharmacological interactions [43]. Furthermore, hypnotherapy has a broad clinical application, and there have been many studies conducted on the efficacy of hypnotherapy in treating many different disorders and symptoms, including insomnia and sleep disorders, acute and chronic pain disorders, and functional neurological disorders [25,44,45]. By appreciating the current evidence surrounding this treatment modality, clinicians in all specialties will be well-informed in using hypnotherapy. Additionally, as this comprehensive review will investigate the impact of hypnotherapy on many different psychiatric symptoms, clinicians will also better understand the efficacy of hypnotherapy in treating various disorders and symptoms, thereby informing their clinical practice.

### Conclusions

This systematic review and meta-analysis protocol outlines a rigorous plan to synthesize the available evidence on the effects of hypnotherapy in addressing burnout and related psychopathologies, including depression, anxiety, and PTSD. By establishing burnout as the primary outcome and related mental health conditions as secondary outcomes, this review aims to clarify the current state of evidence and identify knowledge gaps to inform future research and clinical practice. The findings are expected to guide the design of future trials and help determine whether hypnotherapy represents a promising adjunctive strategy for managing burnout and its associated psychological sequelae.

## Abbreviations

CAPS: Clinician-Administered PTSD Scale
HAM-A: Hamilton Anxiety Rating Scale
HAM-D: Hamilton Depression Rating Scale
MBI: Maslach Burnout Inventory
NHLBI: National Heart, Lung, and Blood Institute
PECOS: Population, Exposure, Comparison, Outcome, Study Design
PICOS: Population, Intervention, Comparison, Outcome, Study Design
PRESS: Peer Review of Electronic Search Strategies
PRISMA: Preferred Reporting Items for Systematic Reviews and Meta-Analyses
PROSPERO: International Prospective Register of Systematic Reviews
PTSD: posttraumatic stress disorder
RCT: randomized controlled trial
